# 2022 ISCB Innovator Award: Núria López-Bigas

**DOI:** 10.1093/bioinformatics/btac336

**Published:** 2022-06-27

**Authors:** Christina Fogg, Diane Kovats, Martin Vingron

**Affiliations:** Freelance Writer, Kensington, MD, USA; International Society for Computational Biology, Leesburg, VA, USA; Max Planck Institute for Molecular Genetics, Berlin, Germany

Each year, ISCB recognizes a scientist who is within two decades of completing her or his graduate degree and has made significant contributions to field of computational biology. The 2022 ISCB Innovator Award winner is Dr. Núria López-Bigas, Group Leader and ICREA Research Professor of the Biomedical Genomics Group at the Institute for Research in Biomedicine, Barcelona, Spain. She will receive her award and give a keynote address at the 30^th^ Conference on Intelligent Systems for Molecular Biology (ISMB) in Madison, WI being held from July 10-14, 2022.

*Núria López-Bigas: Taking Aim at Cancer*


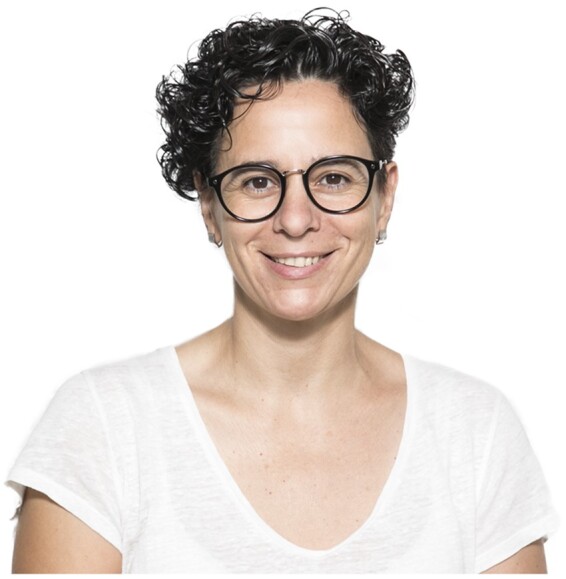


Núria López-Bigas was born in Monistrol de Montserrat, a small town near Barcelona and had very broad interests as a young child, but her inclination toward biology emerged as a high school student, after which she pursued a B.Sc. in Biology at the University of Barcelona. López-Bigas went on to complete her Ph.D. in Biology at the Oncologic Research Institute, University of Barcelona under the mentorship of Xavier Estivill. Her dissertation research focused on the molecular causes of hereditary deafness, and she gained experience tackling questions in Mendelian genetics and validating findings in mouse models of deafness. In 2002, López-Bigas shifted her interest toward computational biology and pursued a postdoc at the European Bioinformatics Institute (EBI) in Cambridge, England under the mentorship of Christos Ouzounis. She recalled the effort that accompanied this shift and said, “It was a different time and there were very few formal training opportunities in bioinformatics, so I had to teach myself how to code.” López-Bigas’s work focused on computational comparative genomics at a time when only a handful of complete genomes existed, and she studied mutations involved in disease, much in the vein of her Ph.D. research on hereditary deafness. She also developed experience in using and analyzing microarrays. López-Bigas was supported by the Human Frontiers Science Program (HFSP) as a postdoc, which supported her research at EBI for two years, then provided her with an opportunity to pursue a project in her home country for the final year. She returned to Spain for this period and joined the laboratory of Roderic Guigó at the Centre for Genomic Regulation in Barcelona, Spain.

López-Bigas began her career as an independent scientist in 2006 and was selected to be a Group Leader and Ramon y Cajal Researcher at the Universitat Pompeu Fabra in Barcelona, Spain. This position was organized as a five-year contract and included only minimal financial support to start her lab, but she also obtained a HFSP Career Development Award which provided some extra funding to get started. López-Bigas recalled, “I started slowly, and focused only on computational biology, no wet lab, and this really helped me make progress.” Her lab studied cancer genetics and they focused on copy number alterations and expression differences because of the availability of arrays and techniques that could measure these genetic features. And then the first cancer genomes were starting to be sequenced. López-Bigas recalled, “It was clear there were lots of mutations in tumors, and we thought it was important to understand how these mutations appear and which ones cause cancer across cancer types.” This research led to her interest in identifying mutations that drive tumorigenesis, and her group has published several pivotal studies detailing how different mutational processes affect specific cells and tissues, and how defects in DNA damage repair pathways alter mutation rates. López-Bigas and her team have also been at the forefront of developing software and data infrastructure for cancer research, including their IntOGen pipeline that has been used to build a compendium of mutational cancer driver genes (www.intogen.org), which provide critical insights into mechanisms that contribute to tumorigenesis. They have also developed the Cancer Genome Interpreter tool that is used to evaluate the biological and clinical impacts of mutations detected in tumor samples and guide treatment (www.cancergenomeinterpreter.org). After the 5 year contract, in 2011, López-Bigas was selected as an ICREA Research Professor, which is a permanent position paid by the Catalan government.

In 2016, López-Bigas and her research group moved to the Institute for Research in Biomedicine (IRB Barcelona). She has expanded her computational biology lab and has built up a wet lab to give her group the ability to generate their own data and not be only dependent on publicly available datasets. With these expanded capabilities, López-Bigas is now using deep sequencing to compare tumor tissue and resected non-tumor tissue from the same patients to gain insight into cancer driver mutations and how small clonal cell clusters might undergo positive selection and turn into tumors.

The COVID-19 pandemic temporarily shut down López-Bigas’s wet lab, but her group was able to continue their computational biology studies since each lab member had a laptop and the ability to connect to the computer cluster. In addition, López-Bigas stepped forward with her team to use their genomics knowledge to help analyze SARS-CoV-2 viral sequences in 2020. She collaborated with scientists in Austria, including Christoph Bock and Andreas Bergthaler, to carry out a genomic epidemiology study of superspreader events and understand factors contributing to mutational dynamics and viral transmission. Members of her team also helped build up the SARS-CoV-2 PCR testing infrastructure in Spain. López-Bigas and her team have now returned full focus on cancer genomics research but were gratified by the opportunities to use their scientific knowledge and skills to help during the pandemic.

López-Bigas has been thankful for her mentorship as a young scientist, which provided her with both intellectual freedom and opportunities to learn new skills. As the mentor of numerous postdocs and students, she has worked hard to create a lab environment where trainees are engaged and collaborative. She said, “As a mentor, I try to get people excited about their projects, motivate them, and encourage collaboration within the lab. An important part of my job is to make the right environment so people can jump into the lab, learn from each other and do interesting science.”

As a leader in the field of computational cancer biology, López-Bigas has served her peers in various capacities, including organizing several meetings related cancer genomics and sequencing for ISMB. She has served as a reviewer of grant proposals and research articles and has been a member of scientific advisory boards of large institutions such as the Finland Institute of Molecular Medicine, the Gustave Roussy Cancer Institute and Open Targets. Her work and service have been recognized by numerous awards and honors, including election as a member of European Molecular Biology Organization (EMBO) and a Fellow of ISCB. She is deeply touched by her recognition with the 2022 ISCB Innovator Award and said, “I was surprised to be selected and humbled and happy. It means the bioinformatics community appreciates and recognizes the work of my group.”

